# Whole-body vibration improves the functional parameters of individuals with metabolic syndrome: an exploratory study

**DOI:** 10.1186/s12902-018-0329-0

**Published:** 2019-01-09

**Authors:** Danúbia Sá-Caputo, Laisa Liane Paineiras-Domingos, Arlete Francisca-Santos, Elzi Martins dos Anjos, Aline Silva Reis, Mario Fritsch Toros Neves, Wille Oigman, Ricardo Oliveira, Andrea Brandão, Christiano Bittencourt Machado, Xavier Chiementin, Redha Taiar, Alessandro Sartório, Mario Bernardo-Filho

**Affiliations:** 1 Programa de Pós-Graduação em Fisiopatologia Clínica e Experimental, Faculdade de Ciências Médicas, Universidade do Estado do Rio de Janeiro, Rio de Janeiro, RJ Brazil; 2 Laboratório de Vibrações Mecânicas e Práticas Integrativas, Departamento de Biofísica e Biometria, Instituto de Biologia Roberto Alcântara Gomes e Policlínica Américo Piquet Carneiro, Universidade do Estado do Rio de Janeiro, Av. 28 de Setembro, 87, fundos, 4° andar, Rio de Janeiro, RJ 20551031 Brazil; 3 Programa de Pós-Graduação em Ciências Médicas, Faculdade de Ciências Médicas, Universidade do Estado do Rio de Janeiro, Rio de Janeiro, RJ Brazil; 4 Faculdade Bezerra de Araújo, Rio de Janeiro, RJ Brazil; 5 Centro Universitário Serra dos Órgãos, Teresópolis, Rio de Janeiro, RJ Brazil; 6 Departamento de Clínica Médica do Hospital Universitário Pedro Ernesto, Faculdade de Ciências Médicas, Universidade do Estado do Rio de Janeiro, Rio de Janeiro, RJ Brazil; 7 Departamento de Cardiologia do Hospital Universitário Pedro Ernesto, Universidade do Estado do Rio de Janeiro, Rio de Janeiro, RJ Brazil; 8 Laboratório de Ultrassom Biomédico, Universidade Estácio de Sá, Rio de Janeiro, Brazil; 9 GRESPI, Moulin de la Housse, Université de Reims Champagne Ardenne, 51687 Reims Cedex 2, France; 10 Experimental Laboratory for Auxo-endocrinological Research, IRCCS, Instituto Auxologico Italiano, 20145 Milan, Italy; 11 Istituto Auxologico Italiano, Laboratorio Sperimentale di Ricerche Auxo-endocrinologiche, IRCCS, Milan, Italy

**Keywords:** Whole-body vibration, Metabolic syndrome, Functional parameters

## Abstract

**Background:**

Metabolic syndrome (MetS) is a cluster of metabolic abnormalities that increases the cardiovascular risk. Regular physical exercise can promote benefits, but the MetS individuals are demotivated to perform it. Thus, new possibilities are important as an alternative intervention. The whole-body vibration can be considered an exercise modality and would be a safe and low-cost strategy to improve functional parameters of individuals in different clinical conditions. The aim of this exploratory study was to assess effects of whole-body vibration on functional parameters of MetS individuals. The hypothesis of this work was that the whole-body vibration could improve the functionality of MetS individuals.

**Methods:**

Twenty-two individuals performed the intervention. The vibration frequency varied from 5 to 14 Hz and the peak-to-peak displacements, from 2.5 to 7.5 mm. Each session consisted of one minute-bout of working time followed by a one minute-bout of passive rest in each peak-to-peak displacement for three-times. The whole-body vibration protocol was applied twice per week for 5 weeks. Data from the trunk flexion, gait speed, sit-to-stand test and handgrip strength were collected. Physiological parameters (blood pressure and heart rate) were also evaluated. The Wilcoxon Rank test and Student t-test were used.

**Results:**

No significant changes (*p* > 0.05) were observed in physiological parameters (arterial blood pressure and heart rate). Significant improvements were found in trunk flexion (*p =* 0.01), gait speed (*p =* 0.02), sit-to-stand test (*p =* 0.005) and handgrip strength (*p =* 0.04) after the whole-body vibration.

**Conclusions:**

In conclusion, whole-body vibration may induce biological responses that improve functional parameters in participants with MetS without interfering in physiological parameters, comparing before and after a 5-week whole-body vibration protocol.

**Trial registration:**

Register in the *Registro Brasileiro de Ensaios Clínicos* (ReBEC) with the number RBR 2bghmh (June 6th, 2016) and UTN: U1111–1181-1177. (virgula).

## Background

According to the International Diabetes Federation (IDF), metabolic syndrome (MetS) is a cluster of metabolic abnormalities that increases the cardiovascular risk. In this context, the insulin resistance and central obesity are considered important factors. Other conditions are relevant in MetS individuals because they can interfere on the management of them, such as i) genetic factors, ii) physical inactivity, iii) aging iv) a proinflammatory state and v) hormonal changes. An increasing risk for type 2 *diabetes mellitus* (T2DM) and cardiovascular disease is observed in MetS individuals [1–4].

**Fig. 1:**
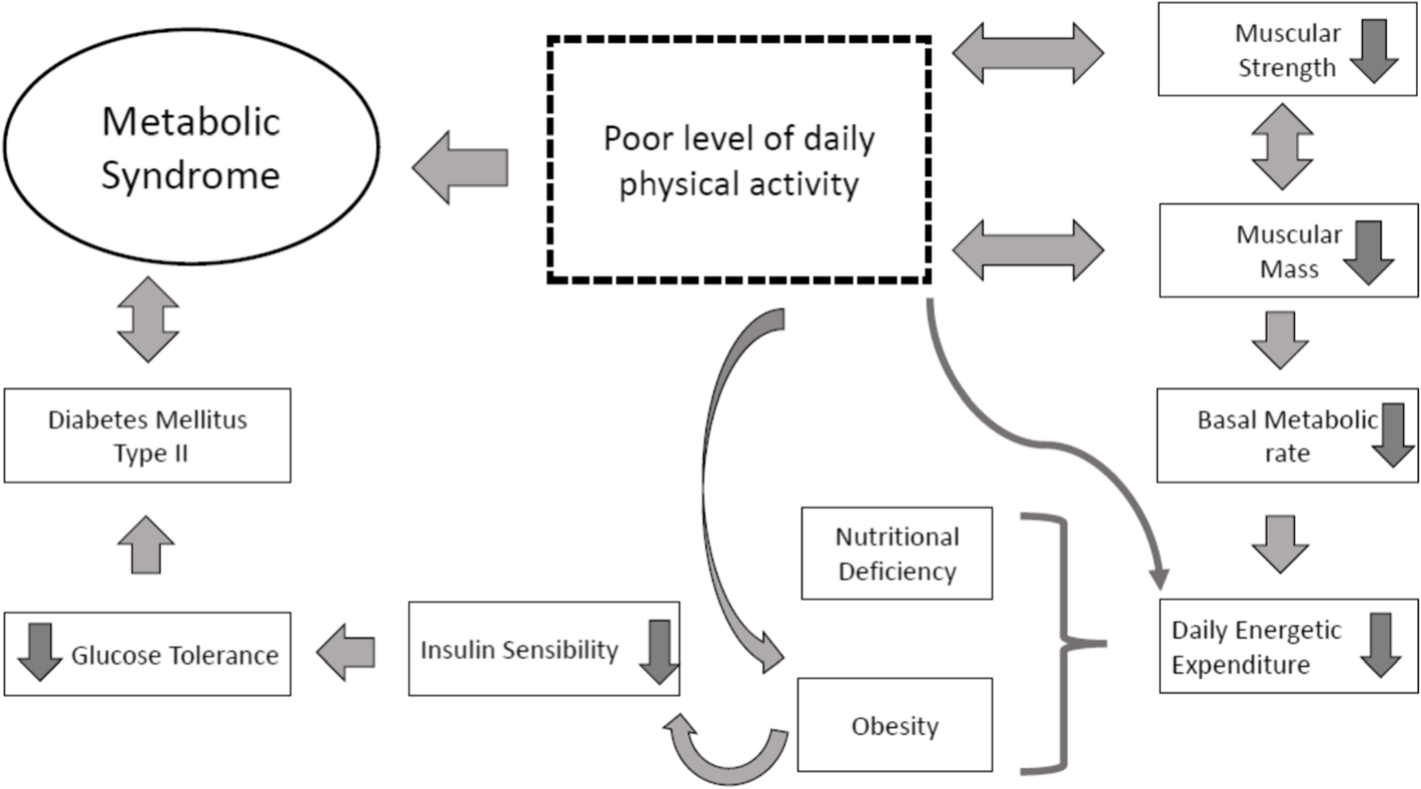
The complex process of the relationship between the poor level daily physical activity and the development of the Metabolic Syndrome

Metabolic impairments caused due to the excessive fat accumulation (central obesity) in obese individuals are associated with increased risk for T2DM, cardiovascular disease, disability and mortality [5, 6]. The central obesity, as observed in MetS participants, negatively affects the function of the insulin receptors within the muscle and is associated with insulin sensitivity through cytokine-mediated pathways [7, 8]. These pathways may help to explain the impairments in the physical function that characterizes the chronic obesity and T2DM and their common complications [9, 10]. This complex process is shown in Fig. 1, where it is verified that poor regular physical activities can lead to MetS. Regular exercise practice, as aerobic exercise, fitness or progressive resistance training exercise, is associated with the improvement of several metabolic parameters, as reduced visceral (abdominal) fat in adults [11] without any change in body mass [12]. It suggests an increase in muscle mass due to exercise training. In a systematic review, Thomas et al., 2006 reported that glycated hemoglobin values decreased after the intervention in physically active groups more than in control groups, in individuals with T2DM [12]. Moreover, Chang et al., 2015 [13] evaluated the body composition, muscular strength, flexibility and cardiorespiratory endurance of community-dwelling elders and it was verified that the presence of MetS was associated with a decrease in flexibility independent on age, gender, and body mass index (BMI).

An exercise modality involving mechanical vibration generated in the oscillating/vibratory platform (OVP) is the whole-body vibration (WBV) [14]. Mechanical vibration, defined as an oscillatory motion, can be generated in OVP and transmitted to a subject [15]. The working time, as well as the rest time between bouts, the number of bouts in a session, and the periodicity of the sessions might be also controlled [15, 16]. Other prescriptive factors of WBV include frequency (*f*), peak-to-peak displacement (*D*) and the position of the individual [17].

WBV can improve the (i) flexibility in male athletes [18], young men [19], MetS individuals [20, 21], young students [22], divers [23], obese sedentary young people [24]; (ii) muscular strength in trained men [25]; (iii) functionality in individuals with multiple sclerosis [26], in MetS individuals [27] and with severe chronic obstructive pulmonary disease [28]; (iv) quality of life in MetS individuals [29] and children with cerebral palsy [30] and (v) gait speed in children with idiopathic toe walking [31]. Considering the publications in the PubMed database, only a limited number of articles involving studies about the effects of the WBV in MetS individuals is found [20, 21, 27, 29].

Milanese et al., 2018 [32] studied the metabolic effect of WBV (short-term) in physically active young males using indirect calorimetry to access the oxygen consumption and they have shown that WBV increases the metabolic cost of exercise that can be complemented with physical activity programs in several settings. Dutra et al., 2016 [33] concluded that low-intensity vibration improves balance, mobility and muscle strength in the upper and lower limbs in postmenopausal women using a 12-month-WBV protocol. Tseng et al., 2016 [34] reported that WBV training at 20 Hz has significant benefit to the balance and flexibility of elderly who do not engage in habitual exercise. Putting together these considerations and the functional limitations of the MetS individuals to perform physical activities, the relevance of the current study was to assess effects of WBV on functional parameters of these individuals.

Therefore, the aim of this exploratory study was to verify effects of whole-body vibration on functional parameters of MetS individuals. A suitable and feasible WBV protocol was applied twice per week for 5-weeks to evaluate the trunk flexion (TF), gait speed (GS), sit-to-stand test (STS) and handgrip strength (HS). Physiological parameters (systolic-SBP, diastolic blood pressure-DBP and the heart rate-HR) were also determined.

The hypothesis of this study was that the WBV could improve the functionality of MetS individuals.

## Methods

### Individuals

In this exploratory study, twenty-eight individuals (61.18 ± 8.39 years old) with MetS were selected to evaluate the effect of the WBV in functional parameters. The recruitment of participants was done from April 2014 to January 2016, made through a screening performed by the medical staff of *Hospital Universitário Pedro Ernesto (HUPE), Universidade do Estado do Rio de Janeiro* (*UERJ*), Brazil. WBV protocol was performed in the *Laboratório de Vibrações Mecânicas e Práticas Integrativas* - LAVIMPI*, UERJ.*

The inclusion criteria were outpatients of both genders, over 40 years old [35] with a previous clinical diagnosis of MetS based on the criteria described by the IDF [1]. The exclusion criteria were individuals with very high blood pressure (≥ 180/110 mmHg) and not controlled, cardiovascular disease (coronary artery disease or stroke), neurological, musculoskeletal or rheumatologic disease does not permit the performance of WBV. Participants who refused to sign the consent form were also excluded.

As the reduction of the flexibility has been also associated with the MetS [13] and could interfere in the functionality of these individuals, this parameter was considered in the determination of the sample size. For a statistical power of 95% and the significance level of 5%, a sample size of 13 participants was calculated to determine a 17% change in flexibility [20, 36]. The Transparent Reporting of Evaluations with Non-randomized Designs (TREND) statements were used to report all the different steps of the interventions utilized in this study [37]. This study was approved by the Research Ethics Committee of the *HUPE, UERJ* with the number CAAE 54981315.6.0000.5259, the register in the *Registro Brasileiro de Ensaios Clínicos* (ReBEC) with the number RBR 2bghmh and UTN: U1111–1181-1177. The principles from the Declaration of Helsinki were followed.

The participants were sedentary, and they were instructed to continue their normal daily activities and medications during the investigation. In general, the medications used by the participants were diuretics, beta blockers, calcium channel blockers, angiotensin-converting-enzyme inhibitors and angiotensin receptor antagonists.

### Anthropometric characteristics

The height and body mass were measured on a digital balance (MIC 200 PPA, Micheletti, São Paulo, Brazil). Then, the BMI was calculated by dividing the mass (kg) by the stature squared (m^2^) [38]. The assessment of the waist circumference (WC) was performed with non-stretchable flexible tapes and the measurement was at the midpoint between the last rib and the iliac crest, according to guidelines by the World Health Organization (WHO) [4].

In an interview, each participant was asked about smoking and physical activity habit and it was considered the answer “yes” or “no”. The participants provided information about hypertension and T2DM diagnosis that the physician had defined (according to IDF).

### Primary outcomes

In this investigation, the findings related to the evaluated parameters before and after a five-week protocol involving WBV were considered. Before the first session, the outcomes were performed in the sequence, physiological parameters, gait speed, sit to stand the test, HS and flexibility with a rest of 10 min among them. After the last session, the measurements were performed in the same way to reduce the measurement errors.

### Determination of physiological parameters

An automated device (OMRON, model HEM-7113, China) was utilized to record the systolic (SBP) and diastolic blood pressure (DBP) (mmHg), and the heart rate (HR) (beats per min – bpm) from the left arm of seated participants after a 10-min rest before each day of WBV [39] and after the WBV session. There was one minute of rest between each measurement. Mean values of three records were used in the analysis.

### Determination of the gait speed

In the evaluation of the GS, the individual was asked to walk on a demarcated distance of 3 m on the floor. Two measures of the walked time (chronometer, cronobio SW2018, Brazil) were obtained, and the best time was considered [40]. The gait speed was calculated dividing the distance by the walked time. This test was performed before and after the WBV protocol.

### Determination of the sit-to-stand test

Measures of gait speed and the sit-to-stand (STS) test have been considered as an important component of physical capacity which gives insight on frailty in older adults [41, 42]. To evaluate the lower-limb functional strength, the STS was used in this current study. The subject was asked to sit in an armless chair (with its back supported against a wall) with arms crossed over his/her chest and then instructed to stand and sit five times as quickly as possible. The same chair was used for all participants.

Participants performed two timed trials (chronometer, cronobio SW2018, Brazil), and the second one was considered for analysis. The instructor started the test with “Ready, Set, Go”, started a digital stopwatch on “Go,” and counted aloud each of the five completed sit to stand cycles. The stopwatch was stopped when the subject returned to the seated position for the fifth time [43]. This test was performed before and after the WBV protocol.

### Determination of the handgrip strength

The muscular strength of the upper limbs (hand) was accessed through the HS, measured quantitatively using a digital hand dynamometer (EMG830RF, EMG System, São José dos Campos, Brazil). This test aims to verify the strength of the upper limbs - the hands specifically. The individuals were positioned sitting down with a straight back and no armrests and with elbow flexion at 90°. They were asked to grip the dynamometer with their dominant hand using maximum strength for 6 s (chronometer, cronobio SW2018, Brazil). The test was performed three times at 1-min intervals, before and after the WBV protocol, and the maximum score, in kgf, was recorded [43]. Handgrip strength has been recommended as an assessment technique for the measurement of muscle strength, and as the simplest method for assessment of muscle function in clinical practice [43]. Authors have reported that HS may be a good predictor of body cell mass depletion and mortality [43, 44].

### Determination of the flexibility

**Fig. 2:**
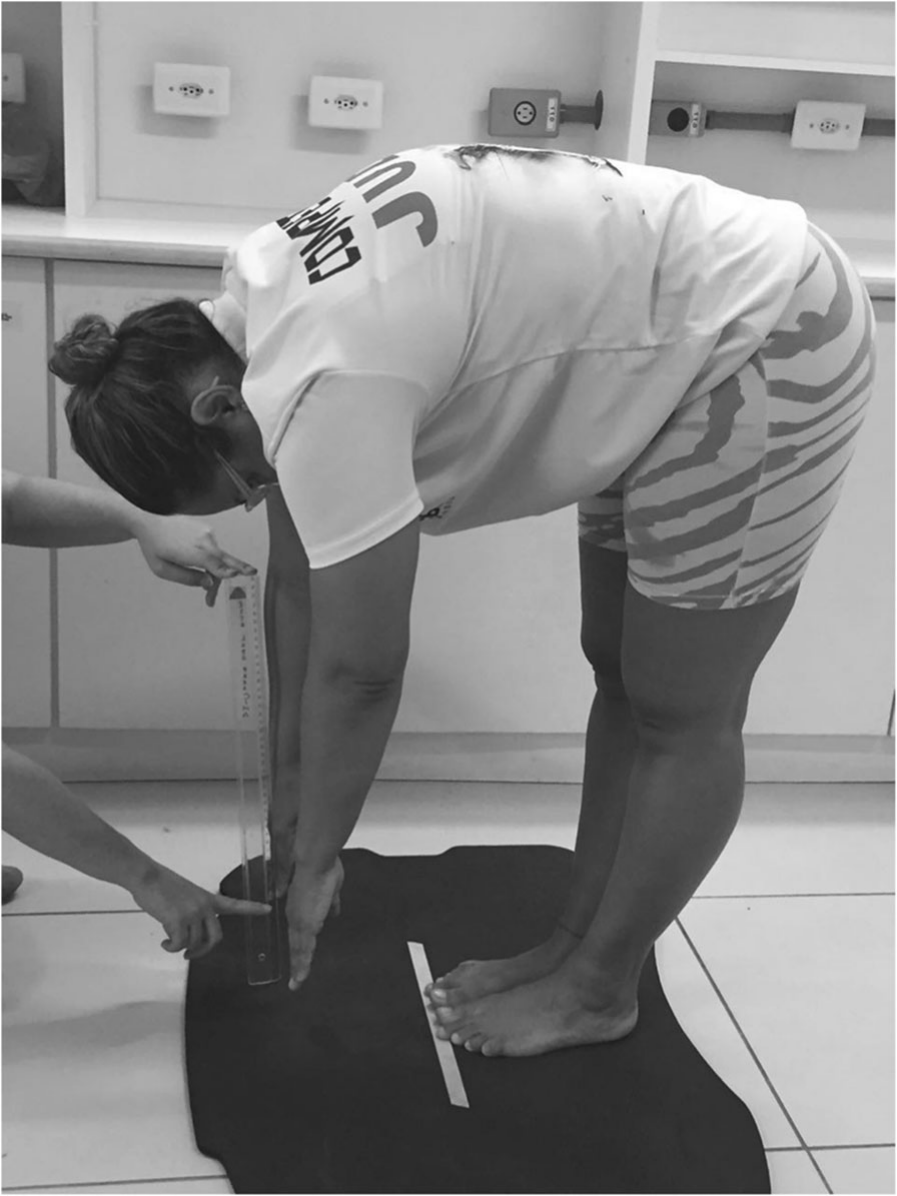
Individual performing the Anterior Trunk Flexion test to measure distance of the third finger and the floor

According to the American Council on Exercise, flexibility is related to the ability to move joints through their full range of motion and it is highly desired and relevant to a subject to do their daily activities [45]. The measure of flexibility was performed through the anterior trunk flexion (ATF) test [20, 46], also called fingertip-to-floor distance (FFD) test. This test consisted in measuring the distance between the tip of middle finger and the floor after an anterior trunk flexion, with feet together and without bending the knees [20] (Fig. 2). The distance between the third was determined before and after of WBV session and it was expressed in centimeters.

### Intervention

Before the first session of the WBV program, the participants were instructed to continue their normal daily activities and medications during the investigation. All the individuals confirmed over the end of the study that the instructions were followed.

The protocol was performed in 5 weeks (10 sessions), with at least 24 h of rest between each WBV session. This protocol has been used in previous studies involving WBV and MetS individuals [20, 21, 27, 29]. Similarly, Milanese et al., 2013 [32] and Sañudo et al., 2013 [47] have used a protocol twice a week.

**Fig. 3:**
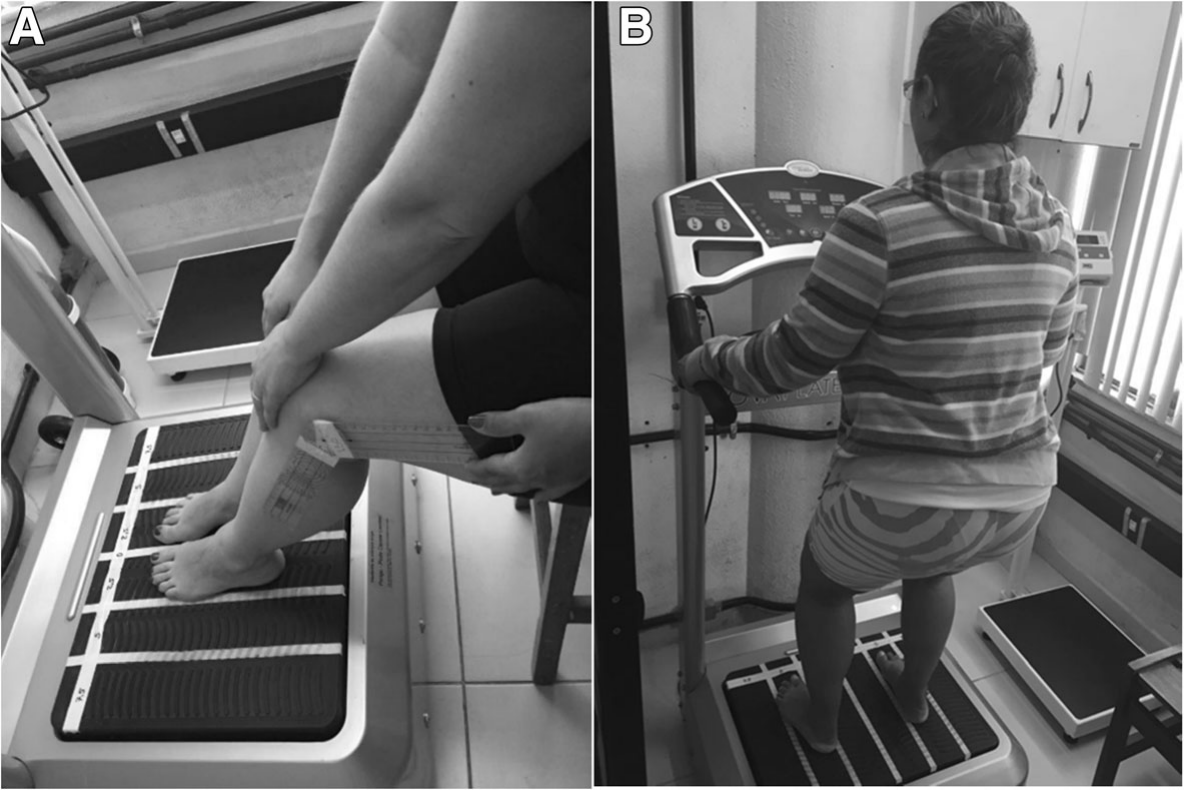
**a**. Participant seated bare feet on a side-to-side alternating oscillating/vibratory platform with knees flexed. **b**. Participant with bare feet in the stand position on the side-alternating platform with knees flexed in a stand position

**Fig. 4:**
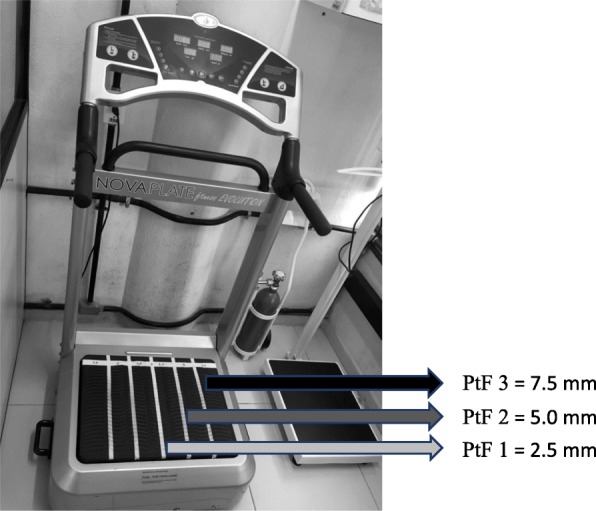
Side-to-side alternating oscillating/vibratory platform used in the study and the positions of the feet (PtF): PtF 1 = 2.5 mm, PtF 2 = 5.0 mm, PtF 3 = 7.5 mm)

In the first session, the individuals sat with bare feet positioned on a side-alternating oscillating/vibratory platform (OVP) (Novaplate, Fitness Evolution ®, São Paulo, Brazil) with knees flexed (130°) controlled by a goniometer [48]. The height of the chair was selected depending on the height of the individual to have similar knee flexion. The hands of the subject were positioned on the knees to facilitate the transmission of the mechanical vibration to the whole body (Fig. 3a). Participants performed three WBV sets of one minute at a frequency of 5 Hz and different peak-to-peak displacements (*D*) for each set. With this WBV machine, the *D* depends on the participant’s foot position. The position of the feet (PtF) on the basis of the platform determines three different *D* (PtF 1–3). The *D* ranged 2.5–7.5 mm and the PtF used were: a) PtF 1–2.5, b) PtF 2–5.0 and c) PtF 3–7.5 mm (Fig. 4). Milanese et al., 2013 [32] used vibration amplitude that ranged 2.0–5.0 mm. Sá-Caputo et al., 2014 [20], Carvalho-Lima et al., 2017 [29], Paineiras-Domingos et al., 2018 [27] and Sá-Caputo et al., 2018 [21] used the same protocol of this current study. For the identification of the different displacements, longitudinal strips of reflective adhesive tape were applied to the OVP. One-minute inter-set time was permitted.

From the second up to the tenth session the individuals were barefooted in a standing position on the side-alternating OVP with knees flexed (130°) [48] Fig. 3b. The hands of the subject were positioned on an appropriate place on the platform and all participants positioned the hands in the same place. Participants performed three WBV sets of one minute with the same *D* described above. One-minute inter-set time was permitted. The total time of the protocol was 17 (9 min of vibration training and 8 min of rest). The frequency in the second session was 6 Hz and 1 Hz was added in the followed sessions until 14 Hz in the tenth session. Similarly, Milanese at al., 2013 [32] have used a protocol with the total time of 19 min (14 min vibration training and 5 min rest) and two vibration amplitude 2.0–5.0 mm. The peak of acceleration was calculated for each set [17].

### Statistical analysis

Descriptive statistics included the mean and standard deviation (SD). The Shapiro-Wilk normality test was calculated to determine if the data set can be modeled as a normal distribution. The Wilcoxon rank test and paired t-test were used to compare the change in the different outcome measures from WBV. The level of significance was set at *p* < 0.05. These analyses were carried out using the Bioestast 5.0 statistic program (*Instituto de Desenvolvimento Sustentável Mamirauá*, Brazil). Values are expressed as mean and standard derivation (SD) in the text. The reliability of the functional tests was assessed by the coefficient variation and the effect sizes were determined (Cohen’s d). Small effect sizes with d ≤ 0.2, moderate effect sizes are 0.2 < d < 0.8, or large effects sizes are d ≥ 0.8 [49] for parametric data, according the formula$$ d=\frac{\mathrm{M}1-\mathrm{M}2}{\sqrt{\begin{array}{c}\mathrm{SD}1+\mathrm{SD}2\ \\ {}2\end{array}}} $$, where M = mean and SD = standard derivation. For non-parametric data were calculated correlation coefficients (*r*) according to the formula *r*$=\frac{Z}{\surd N}$ and reported 0.1 as small effect sizes, 0.3 as moderate effect sizes and 0.5 as large effect sizes [50].

## Results

**Fig. 5:**
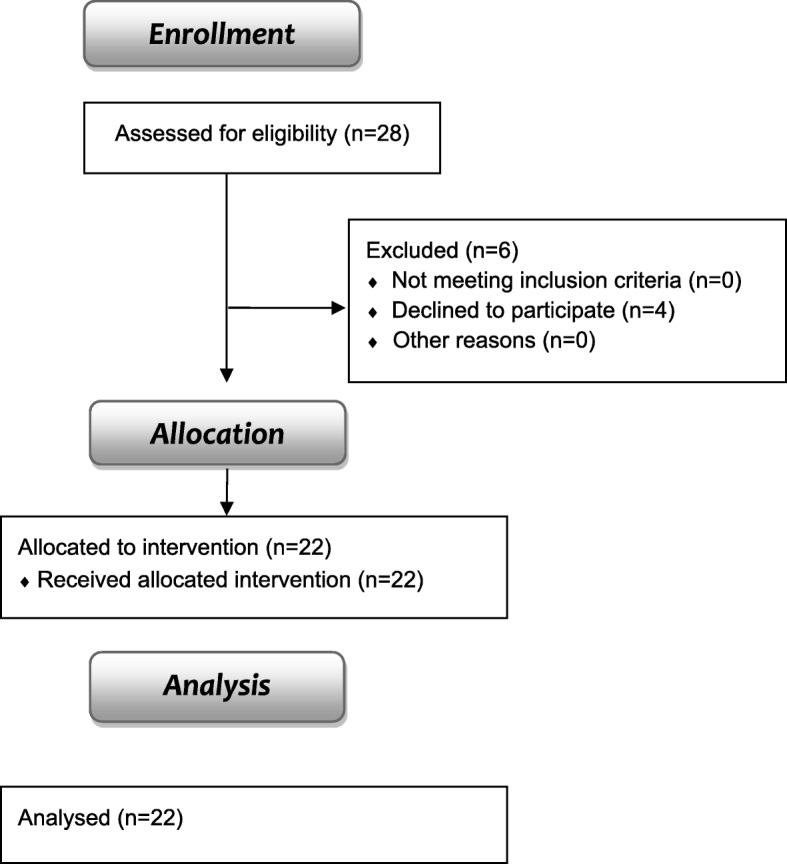
Flow diagram of the clinical intervention

The flow diagram with the enrolment of the study is shown in Fig. 5. Twenty-eight individuals were recruited and six were excluded. Consequently, twenty-two participants (20 women and 2 men) participated in this study. They performed the WBV protocol and the results before the first and after the last session are presented.

The anthropometric characteristics of the participants are presented in Table 1.

**Table 1:** Anthropometric characteristics of the participants

Variables	Mean ± SD or (percentage)
Height (m)	1.63 ± 0.07
Body mass (kg)	83.65 ± 16.27
WC (cm)	103.17 ± 11.09
BMI (kg/m^2^)	31.16 ± 5.35
Smoker (%)	1 (5.26)
T2DM (%)	7 (36.84)
Hypertension (%)	17 (89.47)
Physical activity (%)	4 (21.05)

*WC* waist circumference, *BMI* body mass index, *T2DM* type 2 *diabetes mellitus*

The findings of the physiological parameters as SBP, DBP and HR are presented in Table 2. No significant changes were observed after the WBV with a small effect size (*d* = 0.12), (*d* = 0.15) and (*d* = 0.09) respectively.

**Table 2:** Values of the physiological parameters before and after the WBV protocol

	Before WBV Mean ± SD	After WBV Mean ± SD	*p*-value	Effect-size
SBP (mmHg)	128.93 ± 15.72	126.02 ± 15.10	0.59	*d* = 0.12
DBP (mmHg)	68.08 ± 9.40	66.77 ± 7.93	0.66	*d* = 0.15
HR (bpm)	67.17 ± 13.28	68.37 ± 12.59	0.50	*d* = 0.09

*SBP* systolic blood pressure, *DBP* diastolic blood pressure, *HR* heart rate, *p-* significance at *p* ≤ 0.05, d- Cohen’s d

Concerning the findings of functional parameters (HS, ATF, GS and STS test) before and after the intervention with the WBV protocol, the data are presented in Table 3. It was verified an improvement on the functionality of the individuals with MetS, as it presented.

**Table 3:** Values of functional parameters before and after the WBV protocol

	Before WBV Mean ± SD	After WBV Mean ± SD	*p*-value	Effect-size
HS (kgf)	20.02 ± 7.72	22.41 ± 6.53	0.04	*d* = 0.33
ATF (cm)	16.31 ± 10.31	13.34 ± 11.02	0.01	*r* = 0.60
GS (m/s)	0.73 ± 0.20	0.83 ± 0.27	0.02	*d* = 0.42
STS (s)	20.25 ± 7.74	16.24 ± 3.99	0.005	*r* = 0.65

*HS* handgrip strength, *ATF* anterior trunk flexion – anterior trunk flexion test, *GS* gait speed, *STS* sit-to-stand test; *p-* significance at *p* ≤ 0.05, *d*- Cohen’s d, *r*- correlation coefficients

A significant difference (*p* = 0.005) in the STS test, with a decrease in the time to perform the test, was found, with a large effect size (*r* = 0.65).

Considering the ATF, a significant decrease in this distance (*p* = 0.01), was found with a large effect size (*r* = 0.60). The data about ATF are presented in centimeters.

About the GS, significant (*p* = 0.02) changes, with an increase, were observed after the WBV protocol, with a moderate effect size (d = 0.42).

A significant difference (*p* = 0.04), with an increase in the HS, was found after the WBV protocol, with moderate effect size (d = 0.33).

## Discussion

There is a limited number of publications about the effects of WBV in individuals with MetS [20, 21, 27, 29] involving flexibility, muscular strength, functionality, quality of life and gait speed. The current study demonstrates the possible benefits of WBV to increase the functionality of individuals with MetS. A 5-week (10 sessions) protocol involving WBV improved the functional parameters compared to baseline values. Moreover, physiological parameters were not altered. In addition, the improvement involving the use of WBV might be explained due to WBV can promote coordinated muscle actions [51]. The appropriate muscle responses can occur with correct working of spinal reflex, brain stem balance and cognitive programming [52]. Thus, the muscle stimulus can improve the functionality [53] and adjustment afferent and efferent signals which, in turn, will lead to “training” effects for the sensorimotor system [54]. Studies have described that WBV could reduce the stiffness and hysteresis of the tendon, alter properties of the intramuscular connective tissue and possibly modify those of other passive skeletal structures related to the range of motion for a determined joint [15, 55].

The protocol used in the current study did not alter the physiological parameters (SBP, DBP and HR) of the individuals. These data are important and demonstrate the safety of this protocol with WBV to MetS individuals. Yule et al., 2016 [56] reported that a short-term WBV training with side-alternating OVP did not affect physiological parameters of the patients with chronic stroke. This agrees with the findings reported by Robbins et al., 2014 [57] in which WBV with 40 Hz in a synchronous platform in healthy participants.

Physical limitations, complications and the decrease of the muscle strength of the individuals with MetS can be explained through the relationship between the composition of skeletal muscle tissue and all the affected metabolic pathways [9, 10] (Fig. 1).

Handgrip strength testing is increasingly being used in clinical settings, for example in the assessment of sarcopenia, frailty and undernutrition in hospitalized older people [43]. Sarcopenia, frailty and muscle weakness have been described in individuals with obesity and metabolic disturbs [58] and WBV can be positive effects in these clinical conditions.

WHO reports that physical inactivity favors the reduction of the flexibility [59]. Furthermore, Chang et al., 2015 [13] suggest that exercise interventions to increase flexibility should be implemented to test its possible therapeutic effect on MetS individuals and that the flexibility should be included in the complete evaluation for MetS. As the WBV involves mechanical stretching, this fact could justify the increase of the flexibility by this modality of exercise generated by vibration produced in OVP and the improvements observed in participants exposed to WBV. Sá-Caputo et al., 2014 [20] and 2018 [21]; Gomes and Guimarães, 2004 [60] have reported improvement of the flexibility of participants that have performed WBV. Dallas et al., 2015 [23] examined acute effects of different vibration loads of WBV on flexibility and explosive strength of lower limbs in springboard divers. They reported that WBV is also recommended to increase flexibility and vertical jump height in sports.

In the current study, the mean baseline of the STS test was 20.25 s (Table 3), indicating a somewhat reduced lower-limb muscle strength. Studies have reported an improvement of the lower-limb muscle strength due to WBV in untrained [24] and trained adults [25]. Delecluse et al., 2003 [61] have described that squatting posture during WBV stimulation strengthens the quadriceps muscles, which are knee extensors. The improvement in the STS test time (from 20.25 to 16.24 s), observed in the current investigation may also be related to this WBV stimulation. Williams et al., 2016 [31] described that WBV increases the gait velocity in children with idiopathic toe walking. It could be potentially due to a rapid increase in ankle range of motion or a neuromodulation response.

WBV with the present protocol may improve significantly the functional parameters analyzed, HS, ATF, GS and STS test after the 5-week intervention. The mechanism that explains the effect of WBV on physical function may be a chain of rapid muscle contractions that occur during the exercise. It might directly activate the neuromuscular system in the lower extremities.

Due to the WBV, acute changes occur in endurance-associated parameters such as energy metabolism and turnover [22, 24, 62] and in the neuromuscular activation in the lower extremities [23, 24]. A significant increase in popliteal blood flow (100%) and in the local muscle perfusion in the *gastrocnemius* and *vastus lateralis* muscles were found during and after the WBV [19]. Furthermore, Rittweger, 2000 [15] observed an increase in VO_2,_ dependent on frequency and amplitude and an augmented energy turnover during WBV. Thus, the metabolic and energetic changes occur due to the response to an enhancement in activation intensity in the muscles of the lower extremities during the stimulus promoted by WBV. It could be speculated that the more exposed the muscles are to WBV, the greater the neuromuscular and metabolic demand. Therefore, these findings could aid to understand the improvement of the functional parameters.

The long exposure to WBV and high-frequency vibration seem to have dangerous side effects on the human body [63]. To prevent these potentially dangerous side-effects, it is important to consider safe exercise protocols with a controlled exposure of mechanical vibrations, as the protocol of this investigation. Moreover, side-effects related to WBV were not found in the current study. This could be associated with the controlled parameters used in this protocol with WBV.

A rationale to justify the use of the WBV instead of other activities such as a gym session is due to the simple, feasible, inexpensive, controlled, safe and suitable procedure when performed with the supervision of a qualified professional. Besides various benefits in individuals of different populations, the improvement of the quality of life has been also reported in MetS individuals [29].

There are some limitations in the current study. Firstly, the results (daily activity, daily working, smoking, physical activity and daily energy intake) were not controlled for. Secondly, no long-term follow-up data were available after the intervention, so the long-lasting effects were not investigated. Thirdly, this investigation is an exploratory study and it did not have a control group. Finally, the external validity of this intervention considering its generalizability to other settings (like the everyday-living condition) was not explored. In consequence, further investigations are required.

## Conclusion

In conclusion, WBV may promote an additional effect on local stochastic muscle endurance. It could be speculated that the improvements in local muscle endurance may be caused by adaptations in energy metabolism and turnover, which is associated with vibration-induced changes in neuromuscular activation, that could aid to explain our findings. Moreover, the biological responses to the 5-week WBV protocol would be related to the improvement of the functional parameters on the MetS individuals (HS, ATF test, GS and STS test) without interfering in physiological parameters (SBP, DBP and HR). Further studies are warranted to gain new knowledge about the effects of WBV on MetS individuals.

## Abbreviations

ATF: Anterior trunk flexion test
BMI: Body mass index
*d*: Cohen’s d
*D*: Peak-to-peak displacement
DBP: Diastolic blood pressure
*f*: Frequency
FFD: Fingertip-to-floor distance
GS: Gait speed
HR: Heart rate
HS: Handgrip strength
*HUPE*: *Hospital Universitário Pedro Ernesto*
IDF: International Diabetes Federation
MetS: Metabolic syndrome
OVP: Oscillating/vibratory platform
P_accel_: Peak acceleration
PtF: Position of the feet
*r*: correlation coefficients
ReBEC: *Registro Brasileiro de Ensaios Clínicos*
SBP: Systolic blood pressure
SD: Standard deviation
SE: Standard error
STS: Sit-to-stand test
T2DM: Type 2 *diabetes mellitus*
TREND: Transparent Reporting of Evaluations with Non-randomized Designs statements
*UERJ*: *Universidade do Estado do Rio de Janeiro*
WBV: Whole-body vibration
WC: Waist circumference
WHO: World Health Organization
